# Pleiotropic Effects of Direct Oral Anticoagulants in Chronic Heart Failure and Atrial Fibrillation: Machine Learning Analysis

**DOI:** 10.3390/molecules29112651

**Published:** 2024-06-04

**Authors:** Marco Mele, Antonietta Mele, Paola Imbrici, Francesco Samarelli, Rosa Purgatorio, Giorgia Dinoi, Michele Correale, Orazio Nicolotti, Annamaria De Luca, Natale Daniele Brunetti, Antonella Liantonio, Nicola Amoroso

**Affiliations:** 1 Department of Pharmacy—Drug Sciences, University of Bari, Via Orabona 4, 70125 Bari, Italy; drmelemarco@yahoo.it (M.M.); antonietta.mele@uniba.it (A.M.); paola.imbrici@uniba.it (P.I.); francesco.samarelli@uniba.it (F.S.); rosa.purgatorio@uniba.it (R.P.); giorgia.dinoi@uniba.it (G.D.); orazio.nicolotti@uniba.it (O.N.); annamaria.deluca@uniba.it (A.D.L.); nicola.amoroso@uniba.it (N.A.); 2 Department of Cardiology, “Ospedali Riuniti” University Hospital, Viale Pinto 1, 71100 Foggia, Italy; michele.correale@libero.it; 3 Department of Medical and Surgical Sciences, University of Foggia, Viale Pinto 1, 71100 Foggia, Italy; nd.brunetti@unifg.it; 4 National Institute of Nuclear Physics, Section of Bari, Via Orabona 4, 70125 Bari, Italy

**Keywords:** DOACs, heart failure, atrial fibrillation, machine learning

## Abstract

Oral anticoagulant therapy (OAT) for managing atrial fibrillation (AF) encompasses vitamin K antagonists (VKAs, such as warfarin), which was the mainstay of anticoagulation therapy before 2010, and direct-acting oral anticoagulants (DOACs, namely dabigatran etexilate, rivaroxaban, apixaban, edoxaban), approved for the prevention of AF stroke over the last thirteen years. Due to the lower risk of major bleeding associated with DOACs, anticoagulant switching is a common practice in AF patients. Nevertheless, there are issues related to OAT switching that still need to be fully understood, especially for patients in whom AF and heart failure (HF) coexist. Herein, the effective impact of the therapeutic switching from warfarin to DOACs in HF patients with AF, in terms of cardiac remodeling, clinical status, endothelial function and inflammatory biomarkers, was assessed by a machine learning (ML) analysis of a clinical database, which ultimately shed light on the real positive and pleiotropic effects mediated by DOACs in addition to their anticoagulant activity.

## 1. Introduction

**Figure 1:**
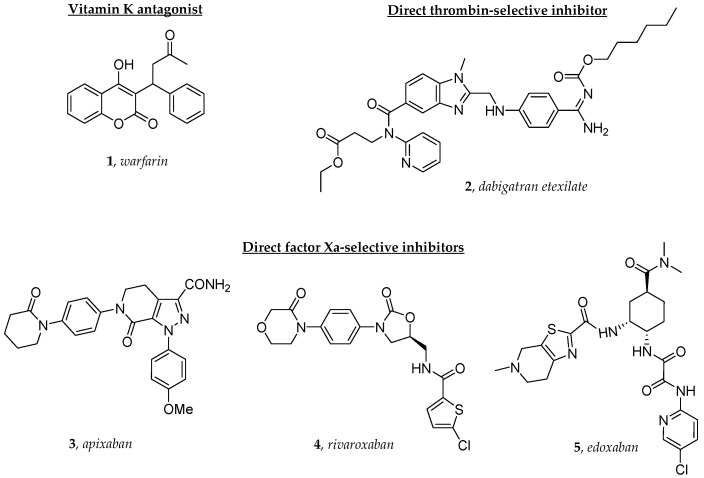
The structures of the oral anticoagulants investigated in this study.

Atrial fibrillation (AF) is a frequent arrhythmia in patients with heart failure (HF). AF prevalence depends on the clinical status of these patients with an incidence that increases along with the severity of HF [1]. The association between AF and HF is easily explainable as these two conditions share the same risk factors, such as arterial hypertension, diabetes mellitus and age. AF in HF patients is associated with increased mortality, stroke and embolism [2,3,4]. The prevention of stroke in AF patients, including those affected by HF, is mainly achieved with oral anticoagulants. In AF patients, the prescription of anticoagulants is usually guided taking into account the balance between ischemic and hemorrhagic risks [5]. Vitamin K antagonists (VKAs), such as warfarin (**1**, Figure 1), provided effective anticoagulation for several decades. However, in the last few years, direct oral anticoagulants (DOACs) proved to be effective and safer alternatives to warfarin in the setting of non-valvular AF [6,7,8]. Unlike warfarin and VKAs, which inhibit the vitamin K epoxide reductase complex 1 (VKORC1), thereby blocking the synthesis of clotting factors, DOACs act as antithrombin-independent selective inhibitors of thrombin (thr), such as dabigatran etexilate (**2**, a double prodrug), or of activated factor X (fXa), such as apixaban (**3**), rivaroxaban (**4**) and edoxaban (**5**), in the blood coagulation cascade (Figure 1). Compared to warfarin, which has a narrow therapeutic index, it has been well established that DOACs provide better safety and efficacy profiles, since they are rapid-onset and short-acting agents that reversibly bind to their targets [9,10]. Their main advantages include simpler clinical use, fewer drug–drug and food–drug interactions, no need for routine monitoring and a lower bleeding risk.

Unlike the three fXa-selective inhibitors **3**–**5**, and warfarin (**1**) as well, the active thr-selective inhibitor dabigatran, which is not orally bioavailable, has been developed and approved for oral administration as a double prodrug (i.e., dabigatran etexilate, **2**) in the mesylate salt form [11]. The prodrug **2** is rapidly converted to the active drug by intestinal and hepatic carboxylesterases 1 and 2 (CES 1 and CES 2), which catalyze the in vivo hydrolysis of the ethyl carboxylate and the *n*-hexyl carbamate moieties, respectively [12].

In a recent large observational study, oral anticoagulants proved to reduce mortality in AF patients in the setting of HF [13]. Moreover, when compared with VKA-based therapy, the use of DOACs was associated with a better outcome in AF hospitalized patients with HF [14] and a lower risk of all-cause mortality in elderly HF patients with AF and renal dysfunction [15]. Interestingly, the switch from VKAs to DOACs in patients suffering from Heart Failure with Reduced Ejection Fraction (HFrEF) and AF was associated with improved endothelial function and C-Reactive Protein levels [16]. Regarding the latter effects, it is conceivable that the actions of DOACs can potentially extend beyond their conventional role in anticoagulation as inhibitors of the blood coagulation factors thr and fXa. Indeed, it is well established that these proteases mediate several (patho)physiological processes such as inflammation, atherothrombosis and angiogenesis [17,18] by triggering the activation of proteinase-activated receptors (PARs). The ability of DOACs to regulate PAR responses provides new insights into their actions beyond anticoagulation. Indeed, several preclinical studies demonstrated that DOACs exhibit pleiotropic actions on endothelial cells such as anti-inflammatory, anti-atherosclerotic and anti-fibrotic effects, as well as the preservation of endothelial integrity [19,20,21,22]. It has been established that DOACs block some pro-inflammatory processes, regulating the expression of some key cytokines in a plethora of in vitro cell systems [22] as well as in animal models of atherosclerotic lesions [23,24]. Furthermore, the decrease in endothelial permeability and reduction in ROS generation have been reported as the main mechanisms underlying the capability of DOACs to enhance endothelial barrier integrity [25,26]. In this context, the relationship between DOAC-mediated pleiotropic effects and clinical efficacy still requires investigation. Moreover, some controversies exist on the use of DOACs in HF patients in the dependence on the single different clinical scenario, and a further in-depth analysis of real word clinical data is highly desirable in this context [27].

Based on the above observations, DOACs may have a positive impact on the prognosis of HF patients with AF, regardless of their anticoagulant effect. Herein, using a machine learning approach, we aim at evaluating the impact of therapeutic switching from warfarin, as representative of VKAs, to DOACs (i.e., dabigatran etexilate, apixaban, rivaroxaban, edoxaban) in patients with HFrEF and AF in terms of cardiac remodeling, clinical status, endothelial function and inflammation biomarkers.

## 2. Results

### 2.1. Patients’ Characteristics

The main clinical characteristics, as well as treatments, of these patients are summarized in Table 1. By using a clustering analysis, we initially evaluated, in the available cohort of 42 patients and based on clinical, biochemical and echocardiographic parameters listed in Table 2, the existence of specific patterns distinguishing the clinical cohorts. Then, by using an RF model, we further investigated the features possibly responsible for the revelation of these two patterns.

### 2.2. Do Clinical Cohorts Show Specific Patterns?

**Figure 2:**
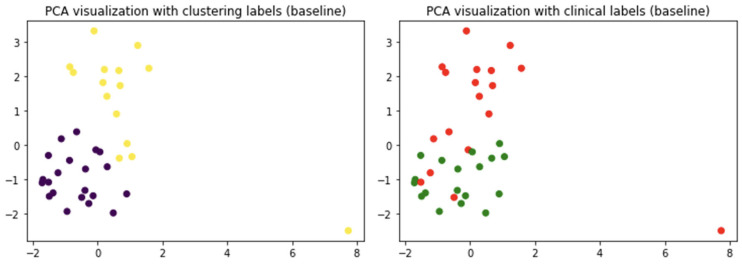
(**left panel**) A PCA visualization of the cluster results: purple and yellow denote the two cluster colors. (**right panel**) Clinical labels: patients who change therapy are in green, DOACs in red. The first two components account for 28% and 27% of the explained variance, respectively.

The first question addressed here concerns the existence of specific patterns distinguishing the clinical cohorts. To this aim, a clustering analysis was performed. The k-means algorithm was able to partially reproduce the clinical cohort. In fact, by inspecting the patient assigned to both classes, at the baseline, 75% of patients were correctly separated. At the follow-up, this accuracy decreased to 64%. Using the first two components of PCA, which account for 55% of the variance, a graphical representation of clusters is presented in Figure 2.

While PCA was used for visualization purposes, it is worth mentioning that the third component accounted for about 16% of the explained variance. The scores’ plot shows at the baseline how the two clusters can be revealed with a limited overlap (left panel). However, inspecting the clinical labels, one can see that these clusters can only yield a partial representation of the clinical cohorts which result in a significant superimposition. To obtain further insight into the informative content provided by PCA, the variance explained was evaluated. In fact, the first two components accounted for 58% of the explained variance. Albeit quite low, the explained variance of the first two PCs highlights a reasonable patient clustering. Moreover, evaluating to what extent the clusters were able to reproduce the clinical labels afforded a cluster purity of about 75%.

**Figure 3:**
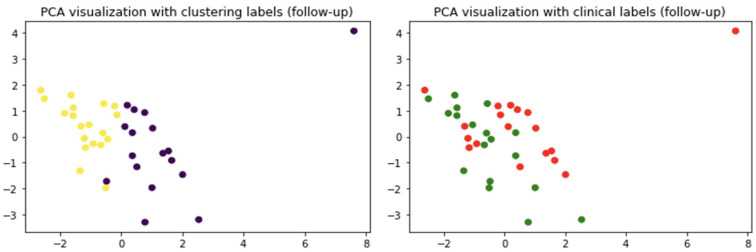
(**left panel**) A PCA visualization of the cluster results: purple and yellow denote the two cluster colors. (**right panel**) Clinical labels: patients who change therapy are in green, DOACs in red. The first two components account for 35% and 23% of the explained variance, respectively.

An analogous analysis was carried out at follow-up, and the results are shown in Figure 3.

In this case, the separation in two clusters appeared less clear. Again, the representation can be considered reasonable as the first two PCs account for about 58% with the third one accounting for about 16% of the explained variance. The clinical cohorts appear overlapped, and this is reflected in the purity measure which decreases to 64%. While at the baseline the clinical cohorts seem to show two distinguished patterns, at the follow-up, these patterns appear to fade.

### 2.3. Outlining the Distinguishing Features of the Clinical Cohorts

**Figure 4:**
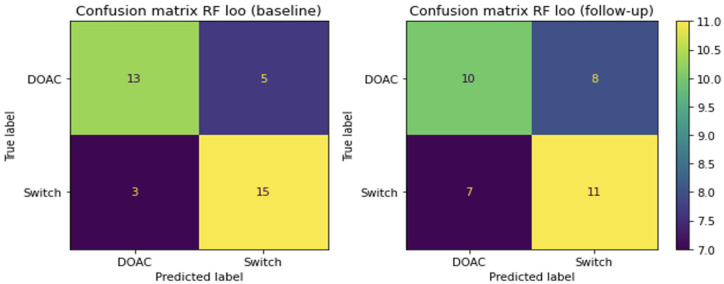
Contingency table for baseline classification (**left panel**). Contingency table for follow-up classification (**right panel**).

Unsupervised clustering revealed the presence, at least at the baseline, of two patterns partially distinguishing the two clinical cohorts. To further inspect this behavior and outline which feature(s) were able to reveal the two patterns, a supervised classification framework was adopted. An RF classifier was used within a loo cross-validation framework to evaluate to what extent the two cohorts were distinguishable. The classification contingency matrices, for both baseline and follow-up, are shown in Figure 4.

**Figure 5:**
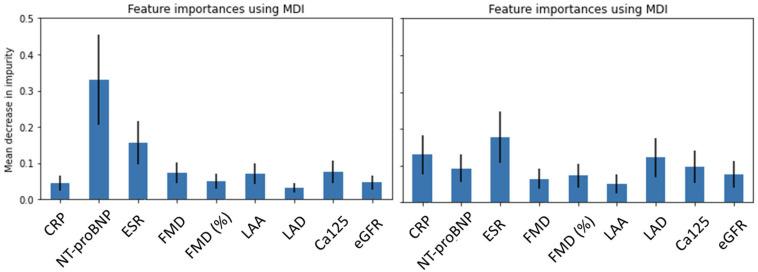
Feature importance measured in terms of mean decrease in impurity (MDI) for both baseline (**left panel**) and follow-up (**right panel**).

The baseline model is more accurate, achieving an average accuracy of 78%. At follow-up, the accuracy decreases to 58%. The accuracy loss of about 20% is statistically significant and suggests a fundamental loss of the features’ discriminative power. To further assess the models’ robustness, cross-validation analyses were repeated 20 times randomly subsampling 30 observations: in this way, we were able to evaluate a 2.5% accuracy uncertainty for both baseline and follow-up results. A feature importance analysis was then performed (Figure 5).

The baseline feature importance shown in the left panel indicates a fundamental contribution in discriminating the clinical cohorts brought by a single feature which is N-terminal pro-brain natriuretic peptide (NTproBNP). This feature alone, at the baseline, results in a mean decrease in impurity of 0.32 with peaks reaching almost 0.5. Of course, it must be kept in mind that RF models combine the available features to fully exploit their informative content; hence, it is not reasonable to assume that one feature is able to distinguish the cohorts on its own; nevertheless, the importance of this feature is manifest. Interestingly, at follow-up, no feature prevails over the others, and the overall importance of all features decreases, not reaching even a 0.2 value of mean decrease in impurity. A “flat” feature importance plot often signals that classification cannot achieve a reliable distinction of the classes.

**Figure 6:**
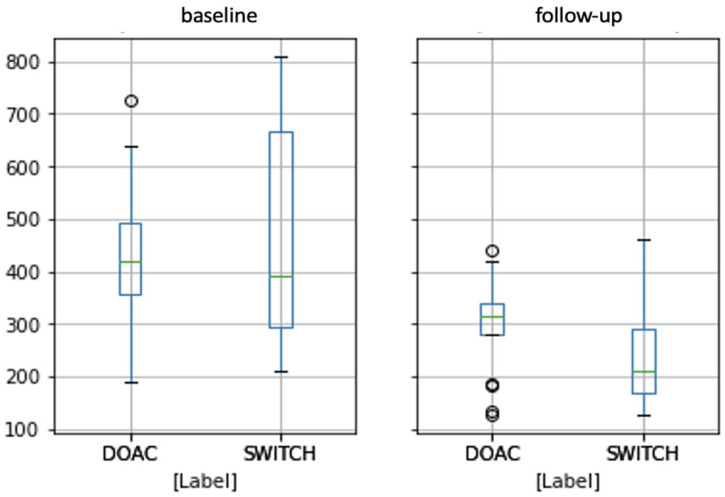
Boxplot representing NTproBNP values at baseline (**left panel**) and follow-up (**right panel**). The green lines represent the distributions’ medians, the circles the statistical outliers.

Finally, to ease clinical interpretation, a comparison of NTproBNP values at baseline and follow-up is provided in Figure 6.

As expected, NTproBNP values decrease for both clinical cohorts, thus demonstrating the efficacy of both treatments. Interestingly, the SWITCH cohort shows a stronger improvement; in fact, while patients treated with DOACs show a decrease in the median NTproBNP from about 420 to 310, the SWITCH cohort shows a decrease of about 200 points, from 400 to 200.

## 3. Discussion

In this study, two groups of patients with AF and HF were analyzed at baseline and at a five-months follow-up: patients already in treatment with DOACs vs. patients switching from warfarin to DOACs. In order to compare these groups, we took into consideration features not directly connected with the anti-thrombotic properties of DOACs but rather related to inflammation, clinical status, endothelial function and cardiac remodeling (Table 2).

Our study demonstrated that patients with AF and HF taking warfarin or DOACs for the prevention of embolic events were clearly distinguishable in terms of remodeling, clinical status, inflammation and endothelial function. Moreover, based on ML analysis, when warfarin-treated patients switched to DOACs, they were no longer differentiable. This means that DOACs somehow modify the considered features which have specific clinical significance (Table 2). Interestingly, a great contribution to this uniformization of VKA and DOACs groups is mainly due to NTproBNP, but the contribution of other features cannot be overlooked.

Brain natriuretic peptide (BNP) and NT-proBNP are secreted in equimolar concentrations into the blood stream after the cleavage of their precursor pro-BNP [28]. Specifically, NT-proBNP performs better than BNP for a longer half-life compared with BNP, and thus, the determination of NT-proBNP levels is today considered the best way to detect the activation of the natriuretic peptide (NP) system [29]. Accordingly, NT-proBNP is a biomarker strictly related to the hemodynamic status of the patient as it is released in response to the stretching of atrial and ventricular walls: in other words, the release of NTproBNP depends on intracardiac pressure and fluid overload [30,31,32]. Factors that cause the stretching of the heart wall can increase the blood level of NT-proBNP. Among the causes of increased heart wall tension, the following can be mentioned: systolic heart failure, the diastolic dysfunction of the heart, restrictive cardiomyopathy, acute coronary syndrome, valvular heart diseases, AF rhythm and amyloidosis [33].

Furthermore, BNP and NT-proBNP are also considered as markers of response to pharmacological treatment. During the treatment, their levels should be checked frequently, and their downward trend indicates a suitable response to the treatment [34]. The lack of a reduction during the treatment indicates an unstable condition and a poor prognosis in the patients. The reduction in NT-proBNP values for both clinical cohorts under investigation in our study also demonstrated the efficacy of DOAC treatment in switched patients.

In general, when a patient switches from VKAs to DOACs, the number of medical contacts is reduced. Thus, the hypothesis that a patient in DOAC treatment is better followed up could be excluded. Moreover, taking into account the features investigated and the observed reduction in NT-proBNP, a positive impact of DOACs, both thr- and fXa-selective inhibitors, on inflammation and endothelial function could be inferred. Indeed, preclinical evidence suggests that nonhemodynamic triggers for natriuretic peptide (NP) release exist, with inflammation increasing the levels of NTproBNP [35,36]. Therefore, inflammatory conditions should be taken into account when interpreting NTproBNP levels, also in the case of patients with HF. NTproBNP could be considered a kind of crosstalk factor between clinical status and inflammatory status specifically associated with patient. Likewise, various indexes of endothelial dysfunction are associated with higher NTproBNP levels [37,38]. Thus, it could be speculated that in our study, the reduction in NTproBNP levels observed in patients treated with DOACs at follow-up could be potentially related to anti-inflammatory effects and the amelioration of endothelial dysfunction mediated by DOACs.

Our study supports previous observations suggesting pleiotropic effects associated with DOACs besides their beneficial action in reducing embolic events and mortality in HF patients. As mentioned above, preclinical studies showed anti-inflammatory, antioxidant and anti-fibrotic effects on endothelial cells. Importantly, several studies highlighted DOAC-mediated effects on endothelial cells in terms of improvement in endothelial function and integrity and the inhibition of neo-angiogenesis [22,39,40]. Although peculiar effects of a single molecule have not yet been determined, it is generally accepted that factor Xa inhibitors produce anti-angiogenic and anti-fibrotic effects beyond the anti-inflammatory action and stabilization of endothelial cells. In line with our study, the authors of [16] demonstrated a protective effect on endothelial function in patients with AF and HF switched from VKA to DOACs.

## 4. Data Sources and Methods

### 4.1. Data Sources

Forty-two consecutive outpatients with CHF and AF in OAT (warfarin or DOACs) for at least one year, enrolled in the Daunia Heart Failure Registry, were followed up between June 2019 and November 2019. At the baseline, within the cohort of DOAC-treated patients, 27.7% were taking dabigatran etexilate, 22.2% apixaban, 33.3% rivaroxaban and 16.6% edoxaban.

**Figure 7:**
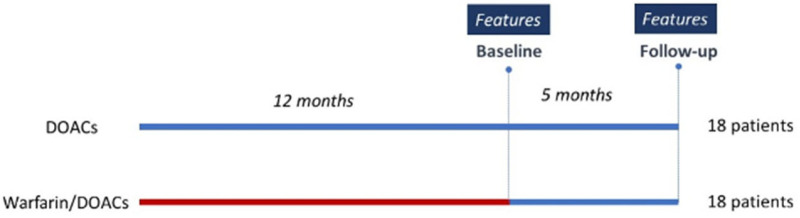
Scheme of patients’ treatment. Blue: DOACs treatment; Red: Warfarin treatment.

Patients shifting from warfarin to DOAC therapy, because of poor patient compliance or time in therapeutic range (TTR), were compared with those already in treatment with DOACs. In the cohort of patients shifted to DOACs, 22.2% took dabigatran etexilate, 44.4% apixaban, 22.2% rivaroxaban and 11.1% edoxaban. All patients underwent an evaluation of clinical status, endothelial function, inflammatory state and cardiac remodeling at the beginning and after 5 months of follow-up (Figure 7).

### 4.2. Method

**Figure 8:**
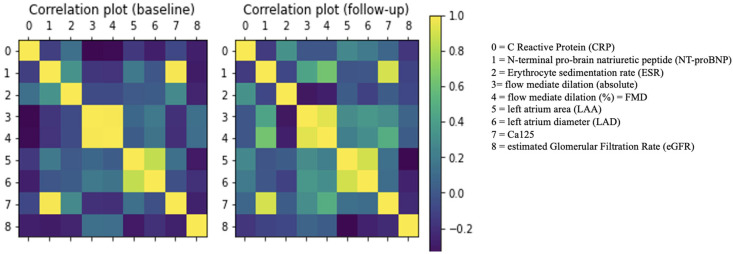
Squared matrix correlation plots of available clinical features. No substantial redundancy can be detected. Max. correlation did not exceed 0.9.

A correlation analysis was performed to evaluate to what extent the clinical descriptors could be considered independent. To this aim, Pearson’s pairwise correlation coefficients were calculated, and the results are shown in Figure 8.

The correlation analysis ensured that no variable had to be excluded; in fact, no correlation exceeded the 0.9 value which is commonly adopted as a threshold value to identify high-correlated variables.

#### 4.2.1. Clustering Analyses

To evaluate how switching from warfarin to DOACs may affect the clinical progression of the patients, we carried out two separate analyses. The case query is whether or not changing therapy has possible drawbacks or, on the contrary, it can even trigger an improvement in patients’ clinical conditions. First of all, carrying out a clustering analysis, the existence of patterns distinguishing the clinical cohorts was examined. In fact, clustering allows us to determine if, according to the examined clinical features, it is possible to classify the patients in different groups. Of course, given the underlying assumption that switching from one treatment to another should not be detrimental for patients, this analysis should assess whether a substantial overlap between the cohorts exist or not.

The fundamental idea behind clustering techniques is that each patient can be seen as a point in the multidimensional space defined by the examined clinical features; accordingly, it is possible to measure the pairwise distances among all patients enrolled and define the so-called similarity/dissimilarity matrix. Then, several approaches can be adopted to separate the data points in different classes. Hierarchical clustering techniques outline the possibility to segregate data in a varying number of classes; thus, they are particularly useful when it is not possible to establish the number of classes a priori or in cases where multiple classifications can coexist. Here, a centroid-based approach was adopted because the number of desired classes is already known, i.e., the class of patients treated with DOACs and the class of patients switching from warfarin to DOACs.

Herein, the k-means clustering method was adopted [41]. According to this specific analysis, two random points, representing the centroids of clusters, were randomly initialized in the features’ space, and then the k-means algorithm proceeded to assign each data point, i.e., each patient, to one class or another according to the distance from the centroids. Of course, this clustering approach is highly sensitive to the initialization, and therefore, we performed one hundred different simulations to establish the average clusters.

Then, to visualize the clustering results, a data reduction technique was adopted. As no assumption can be made about the clinical data, a standard Principal Component Analysis (PCA) was performed. The first two components were suitably used to visualize the clustering results and ultimately evaluate if these clusters reproduce the clinical cohorts. This approach may provide only qualitative evaluations. Therefore, a second analysis, based on machine learning, was carried out to quantify the possible overlapping between the patients treated with DOACs and those switching from warfarin.

#### 4.2.2. Random Forest Classification

Random Forest (RF) is a supervised learning algorithm which, based on the labels, tries to separate the sample observations [42]. The reason behind the evocative name is because this classifier is basically an ensemble of simple classification trees. The ensemble of trees, usually referred to as the forest, provides a classification by means of majority voting. Each tree is grown using a random subsample of available training data; additionally, at each split of the tree (the leaves), a random subsample of features is used to separate the labeled classes. This injection of randomness is the reason behind the algorithm’s name. The advantage is to deliver a model which is accurate and robust to overfitting. This algorithm is particularly adopted because of its light computational cost and the ease of tuning, as it only depends on two major parameters which are the number of trees used to grow the forest and the number of features used at each leaf.

Another fundamental advantage of RF models is their interpretability. In fact, like all tree-based models, RF provides a simple feature importance evaluation based on the purity of leaves obtained using specific features. The more a feature provides pure leaves, the greater its importance. Accordingly, RF provides both an accurate model to distinguish the available classes and a feature importance ranking which outlines how the classification results were achieved. In this sense, it is an optimal choice for clinical purposes. All the analyses were carried out with Python 3.9 and its open source libraries numpy, pandas and scikit-learn. For the presented analyses, the RF standard configuration with 100 trees was adopted; moreover, for the sake of reproducibility, a random seed of 0 was set.

Given the limited sample size, a leave-one-out (loo) cross-validation framework was adopted. Although loo cross-validation has some issues, the most important one being the risk of overestimating the model’s predictive performance, it remains the best choice when the data amount is limited. In fact, in this framework, all but one observation is used to learn the model, while the one left out is used for validation purposes. In this way, especially when the amount of data is limited, the number of observations used to learn the model is maximized. Here, the adoption of machine learning is aimed at providing a measure of the separation between the available patients’ classes, and therefore, the possible performance overestimation, which could cause problems when testing the model on unseen data, does not represent an issue.

## 5. Conclusions

Our ML analysis demonstrated that in a population of patients with HF and AF, patients treated with the VKA warfarin or DOACs are distinguishable according to features related to inflammation, endothelial function, cardiac remodeling and clinical status. Due to the clinical database composition, our multivariate analysis cannot distinguish the behavior of a selective thrombin inhibitor (dabigatran etexilate) from that of selective inhibitors of factor Xa (apixaban, rivaroxaban and edoxaban). It appeared quite clear, however, that the switch from warfarin to DOACs, regardless of their pharmacological target in the blood coagulation cascade and their pharmaceutical properties, aligned the features of the patients, particularly in relation to NTproBNP levels.

## Figures and Tables

**Table 1:** List of clinical characteristics and treatments of patients included in this study.

Clinical Features		Medications	%
Age (years)	70.6 ± 1.5	ACEi/ARB/ARNI	92.8
Male (%)	64.3	Beta-blockers	78.5
Body weight (kg)	74 ± 1.5	MRA	40.5
SBP (mmHg)	110 ± 2.0	Diuretics	54.7
DBP (mmHg)	66 ± 1.7	Ivabradine	2.4
Heart rate (bpm)	64 ± 1.1	Digoxin	9.7
LVEF (%)	47 ± 1.8	Amiodarone	31.7

Abbreviations: angiotensin-converting enzyme inhibitor, ACEi; angiotensin II receptor blocker, ARB; angiotensin receptor–neprilysin inhibitor, ARNI; beats per minute, bpm; diastolic blood pressure, DBP; left ventricular ejection fraction, LVEF; mineralcorticoid receptor antagonist, MRA; systolic blood pressure, SBP.

**Table 2:** List of clinical, biochemical and echocardiographic parameters used for clustering and RF analysis.

Clinical Significance	Feature	Value
**Index of inflammation**		
state of systemic inflammation	CRP (mg/L)	1.7 ± 0.1
	ESR (mm/h)	30.0 ± 1.9
**Index of clinical status**		
congestion/fluid overload	NTproBNP (pg/mL)	652 ± 190
	Ca125 (U/mL)	34.0 ± 11.6
kidney function	eGFR (mL/min)	62.8 ± 3.3
**Index of endothelial function**		
vasodilation in response to increased blood flow	FMD (%)	11.8 ± 1.78
**Index of cardiac remodeling**		
left atrial size	LAD (mm)	44.6 ± 1.0
	LAA (mm^2^)	24.0 ± 0.7

The value reported refers to baseline characteristics. Abbreviations: C-Reactive Protein, CRP; Erythrocyte Sedimentation Rate, ESR; estimated Glomerular Filtration Rate, eGFR; Flow-Mediated Dilation, FMD; left atrial area, LAA; left atrial diameter, LAD; plasma N-terminal pro-brain natriuretic peptide, NT-pro-BNP.

## Data Availability

Data are contained within the article.
